# The prognostic role of C-reactive protein to albumin ratio and anti-MDA5 antibody-positive in idiopathic inflammatory myopathy: a retrospective study

**DOI:** 10.1038/s41598-023-30595-y

**Published:** 2023-03-08

**Authors:** Pei Zhou, Qinxue Shen, Shiting Zhou, Xiaoli Ouyang, Ting Guo, Min Song, Wei Guo, Yi Zhang, Hong Peng

**Affiliations:** 1 Department of Respiratory and Critical Care Medicine, the Second Xiangya Hospital of Central-South University, Changsha, Hunan China; 2 Research Unit of Respiratory Disease, Central-South University, Changsha, Hunan China; 3 The Respiratory Disease Diagnosis and Treatment Center of Hunan Province, Changsha, Hunan China

**Keywords:** Diseases, Risk factors

## Abstract

This cohort study aimed to identify the characteristics and risk factors of adult idiopathic inflammatory myopathy-associated interstitial lung disease (IIM-ILD) and further explore the prognostic factors of IIM-ILD. We extracted data regarding 539 patients with laboratory-confirmed idiopathic inflammatory myopathy (IIM) with or without interstitial lung disease (ILD) from the Second Xiangya Hospital of Central South University between January 2016 and December 2021. The regression analysis was conducted to identify the possible risk factors for ILD as well as mortality. Of 539 IIM patients, 343 (64.6%) were diagnosed with IIM-ILD. The median (IQR) baseline neutrophil-to-lymphocyte ratio (NLR), C-reactive protein to albumin ratio (CAR) and ferritin were 4.1371 (2.6994–6.8143), 0.1685 (0.0641–0.5456) and 393.6 (210.6–532.2), respectively. Risk factors associated with IIM-ILD were older age (*p* = 0.002), arthralgia (*p* = 0.014), lung infection (*p* = 0.027), hemoglobin (*p* = 0.022), high CAR (*p* = 0.014), anti-aminoacyl-tRNA synthetase (anti-ARS) antibody-positive (*p* < 0.001), and anti-MDA5 antibody-positive (*p* < 0.001). The IIM-ILD patients whose age at diagnosis of disease ≥ 59.5 (HR = 2.673, 95% CI 1.588–4.499, *p* < 0.001), NLR ≥ 6.6109 (HR = 2.004, 95% CI 1.193–3.368, *p* = 0.009), CAR ≥ 0.2506 (HR = 1.864, 95% CI 1.041–3.339, *p* = 0.036), ferritin ≥ 397.68 (HR = 2.451, 95% CI 1.245–4.827, *p* = 0.009) and anti-MDA5 antibody-positive (HR = 1.928, 95% CI 1.123–3.309, *p* = 0.017) had a higher mortality rate. High CAR and anti-MDA5 antibody-positive are more likely to be associated with a high mortality rate of IIM-ILD, which can be used as serum biomarkers, especially the CAR, a simple, objective tool to assess the prognosis of IIM.

## Introduction

Idiopathic inflammatory myopathy (IIM) is an umbrella term encompassing a set of disorders, most, but not all, of which are characterized by **c**hronic muscle inflammation. The clinical presentation of IIM is varied and can affect other organ systems including the skin, lungs, and joints. According to most guidelines and expert consensus, the five major types of IIM include dermatomyositis (DM), polymyositis (PM), anti-synthetase syndrome (ASS), immune-mediated necrotizing myopathy (IMNM), inclusion body myositis (IBM)^1^.Interstitial lung disease (ILD) is one of the most common pulmonary manifestations of IIM that affects the interstitial space, the primary consequence of which is impaired gas exchange, thus giving rise to breathlessness, respiratory failure, and even death in IIM patients^2–5^. ILD has an estimated prevalence of 20–78% among the IIM population^3,6–8^, and the median survival of idiopathic inflammatory myopathy-associated interstitial lung disease (IIM-ILD) patients is usually 3–5 years^9,10^. To date, the survival time of IIM-ILD is longer than before, which may be related to the improving awareness and multimodality therapy of this disease. Once diagnosed with rapidly progressive ILD (RP-ILD), patients may carry a poor prognosis with a median untreated life expectancy from diagnosis of 6 months or 1 year^11^. The high morbidity and mortality have urged us to look for simple and convenient biomarkers that can predict the prognosis of IIM-ILD^12^.

Therefore, we sought to identify baseline factors associated with ILD and explore indicators that can be used as independent prognostic biomarkers in patients with IIM-ILD, which will better inform clinical decision-making for the patients.

## Method

### Patients

This was a retrospective cohort study. All subjects were admitted to the Second Xiangya Hospital of Central South University from January 2016 to December 2021. The eligibility criteria for IIM patients were: (1) Age at disease onset ≥ 18 years; (2) Diagnosis based on the criteria for IIM^4,13–15^. (3) The data of myositis-specific autoantibodies (MSA) and myositis-associated autoantibodies (MAA) detected using the EUROLINE Autoimmune Inflammatory Myopathies 16 Ag (IgG) commercial line blot test was complete. IIM complements other systemic autoimmune diseases such as systemic lupus erythematosus (SLE), Sjogren’s syndrome (SS), and systemic sclerosis (SSc) were excluded. The diagnosis of ILD was based on the 2013 American Thoracic Society/European Respiratory Society (ATS/ERS) criteria^16^.

The study was approved by the ethics review committee of the Second Xiangya Hospital of Central South University (No. LYF2022147), and the requirement for informed consent was waived by the aforementioned research ethics board due to the retrospective nature of the study. This study was conducted in accordance with the Declaration of Helsinki.

### Data collection

We extracted demographics and general information including the department of consultation, age at diagnosis, duration of disease, history of smoking, therapy before and after admission, comorbidities and symptoms. Laboratory findings, such as blood cell counts, albumin (ALB), globulin (GLO), C-reactive protein (CRP), erythrocyte sedimentation rate (ESR), lactate dehydrogenase (LDH), creatine kinase (CK), creatine kinase MB (CKMB), myohemoglobin (MB), Neutrophil-to-lymphocyte ratio (NLR), platelet-lymphocyte ratio (PLR), monocyte-lymphocyte ratio (MLR), the CRP to ALB ratio (CAR), the ESR to ALB ratio (EAR), ferritin and serologic autoantibodies were all collected. We also analyzed the imaging findings, such as irregular linear opacities, grid shadow, nodular shadow, septal thickening, ground-glass opacities, patch shadow, traction bronchodilation, honeycombing, and pleural effusion. It is worth emphasizing that an abnormal chest CT scan was defined as any ILD meeting the 2013 American Thoracic Society criteria^16^. High-resolution CT (HRCT) scans were reevaluated independently by two experienced chest radiologists.

### Definition

NLR and PLR were calculated using hematological parameters (absolute count of neutrophil, lymphocyte and platelet) measured by automated analyzers: NLR = absolute neutrophil count (ANC)/absolute lymphocyte count (ALC) and PLR = absolute platelet count (APC)/ALC. The CAR was calculated as CAR = serum CRP level (mg/L)/serum albumin level (g/L), EAR = serum ESR level (mm/h)/serum albumin level (g/L).

### Outcomes and follow-up

The primary endpoint of the IIM-ILD cohort was all-cause mortality. Survival was defined as the interval between the diagnosis of IIM-ILD and death or the latest updated data (June 30, 2022).

### Statistical analysis

All data were analyzed using SPSS 25.0 software (SPSS Inc., Chicago, IL, https://www.ibm.com/cn-zh/spss) and GraphPad Prism 9.0 (GraphPad Software, https://www.graphpad-prism.cn/). Comparisons between groups of continuous variables were made using T-test or the Mann–Whitney test. Comparisons between groups of categorical variables were made using the Chi-square test or Fisher’s exact when suitable. Logistic regression analysis was used to choose candidate risk factors of ILD. The Kaplan–Meier curves with log-rank tests were employed to access predictors in survival. The optimal cut-off values of the potential biomarkers were determined using the receiver operating characteristic (ROC) analysis. A two-tailed *p* < 0.05 was considered statistically significant, and the odds ratio (OR) or hazard ratio (HR) and 95% confidence interval (CI) were indicated.

## Result

### Baseline characteristics of patients with IIM-ILD and IIM without ILD

Of the 539 IIM patients enrolled in this study, 309 (57.3%) were diagnosed with DM and 107 (19.9%) were diagnosed with PM, and other patients were diagnosed with ASS or IMNM. The baseline characteristics of the IIM patients are shown in Table 1. A total of 62.5% presented to the Rheumatology and Immunology Department, and 62.3% of patients were female. Among the IIM patients, 343 (63.6%) had ILD. The mean age of IIM-ILD patients was older than that of the IIM without ILD (IIM-NILD) patients (*p* = 0.017). Concerning clinical symptoms, IIM-ILD patients showed more respiratory and joint manifestations (*p* < 0.001), and pulmonary signs (*p* < 0.001). Moreover, the IIM-ILD group was associated with more Gottron’s rash, mechanic’s hand, dry mouth, fever, hair loss, and weight loss. In contrast, the IIM-NILD group showed a higher percentage of dysphagia (*p* = 0.014). It is worth mentioning that the IIM-NILD group was more likely to combine tumors and tuberculosis, while the IIM-ILD group usually had comorbid lung infection and respiratory failure. Additionally, no statistically significant baseline differences were observed in the duration of the disease and Raynaud’s phenomenon (Table 1).

**Table 1:** Comparison of clinical characteristics between IIM-ILD group and IIM-NILD group.

Characteristic	All patients	IIM-ILD	IIM-NILD	*p*-value
Number	539	343 (63.6)	196 (36.4)	
Age at diagnosis (years)	52.6 ± 12.4	53.7 ± 11.1	50.8 ± 14.2	0.017
Duration of disease, (years)	3 (2–12)	3 (2–11)	4 (2–12)	0.253
Female	336 (62.3)	216 (63.0)	120 (61.2)	0.687
Current smokers	55 (10.2)	32 (9.3)	23 (11.7)	0.272
Diagnosis				< 0.001
Dermatomyositis	309 (57.3)	192 (56.0)	117 (59.7)	
Polymyositis	107 (19.9)	41 (12.0)	66 (33.7)	
Department				< 0.001
Respiratory	79 (14.7)	76 (22.2)	3 (1.5)	
Rheumatology	337 (62.5)	220 (64.1)	117 (59.7)	
Dermatology	87 (16.1)	33 (9.6)	54 (27.6)	
Signs				
Thick breath sounds	109 (20.2)	96 (28.0)	13 (6.6)	< 0.001
Low breath sounds	41 (7.6)	36 (10.5)	5 (2.6)	< 0.001
Moist rales	202 (37.5)	193 (56.3)	9 (4.6)	< 0.001
Clinical manifestation				
Respiratory symptoms				
Cough	226 (41.9)	194 (56.6)	32 (16.3)	< 0.001
Expectoration	165 (30.6)	140 (40.8)	25 (12.8)	< 0.001
Chest tightness	61 (11.3)	50 (14.6)	11 (5.6)	0.001
Dyspnea	247 (45.8)	208 (60.6)	39 (19.9)	< 0.001
Skin manifestations				
Gottron’s rash	221 (41.0)	156 (45.5)	65 (32.2)	0.003
Raynaud’s phenomenon	35 (6.5)	26 (7.6)	9 (4.6)	0.176
Mechanic’s hand	31 (5.8)	29 (8.5)	2 (1.0)	< 0.001
Joint manifestations				
Arthralgia	159 (29.5)	129 (37.6)	30 (15.3)	< 0.001
Joint tenderness	75 (13.9)	62 (18.1)	13 (6.6)	< 0.001
Swollen joint	69 (12.8)	57 (16.6)	12 (6.1)	< 0.001
Muscle manifestations				
Myalgia	215 (39.9)	138 (40.2)	77 (39.3)	0.829
Myasthenia	408 (75.7)	253 (73.8)	155 (79.1)	0.166
Dysphagia	100 (18.6)	53 (15.5)	47 (24.0)	0.014
Others				
Dry mouth	67 (12.4)	56 (16.3)	11 (5.6)	< 0.001
Fever	111 (20.6)	95 (27.7)	16 (8.2)	< 0.001
Hair loss	43 (8.0)	35 (10.2)	8 (4.1)	0.012
Weight loss	211 (39.1)	156 (45.5)	55 (28.1)	< 0.001
Comorbidities				
Tumor	39 (7.2)	15 (4.4)	24 (12.2)	0.007
Lung infection	192 (35.6)	151 (44.0)	41 (20.9)	< 0.001
Tuberculosis	39 (7.2)	17 (5.0)	22 (11.2)	0.006
Hypertension	91 (16.9)	59 (17.2)	32 (16.3)	0.794
Diabetes	52 (9.6)	30 (8.7)	22 (11.2)	0.349
Dyslipidemia	251 (46.6)	153 (44.6)	98 (50.0)	0.227
Respiratory failure	54 (10.0)	50 (14.6)	4 (2.0)	< 0.001
Laboratory results				
Hemoglobin (g/L)	122.9 ± 17.5	121.7 ± 16.9	125.0 ± 18.4	0.040
Monocyte (*10^9^/L)	0.40 (0.27–0.56)	0.39 (0.27–0.56)	0.40 (0.30–0.57)	0.512
NLR	4.6406 (2.9200–7.5789)	4.7794 (3.0109–7.9672)	4.5174 (2.8325–7.0896)	0.237
PLR	207.32 (139.67–306.67)	218.50 (149.83–316.82)	183.88 (127.79–288.19)	0.013
Albumin (g/L)	32.7 ± 6.2	31.3 ± 5.9	35.1 ± 6.1	< 0.001
Globulin (g/L)	29.1 (25.7–33.7)	30.4 (26.7–34.3)	27.1 (24.3–31.2)	< 0.001
Creatine kinase (U/L)	257.10 (64.5–1777.5)	176.6 (55.5–975.9)	670.5 (113.8–3104.9)	< 0.001
Myohemoglobin (U/L)	161.7 (61.2–900.1)	122.4 (50.9–589.5)	315.3 (89.6–1464.1)	< 0.001
CRP (mg/L)	5.45 (2.13–16.46)	7.10 (3.04–19.51)	3.46 (1.48–9.68)	< 0.001
ESR (mm/h)	27 (14–46)	33 (17–51)	20 (11–33)	< 0.001
CAR	0.1685(0.0641–0.5456)	0.2149(0.0890–0.7233)	0.1000(0.0400–0.2845)	< 0.001
EAR	0.8357 (0.4132–1.4894)	1.0638 (0.5523–1.7818)	0.5822 (0.3166–1.0201)	< 0.001
Ferritin (ng/mL)	393.6 (210.6–532.2)	437.2 (279.9–562.1)	313.9 (136.4–442.7)	< 0.001
Myositis associated autoantibodies				
Anti-Ro-52 antibody	289 (53.6)	213 (62.1)	76 (38.8)	< 0.001
Myositis specific autoantibodies				
Anti-Mi-2α antibody	14 (2.6)	4 (1.2)	10 (5.1)	0.006
Anti-Mi-2β antibody	46 (8.5)	28 (8.2)	18 (9.2)	0.683
Anti-TIF1γ antibody	67 (12.4)	27 (7.9)	40 (20.4)	< 0.001
Anti-MDA5antibody	182 (33.8)	162 (47.2)	20 (10.2)	< 0.001
Anti-NXP2 antibody	27 (5.0)	12 (3.5)	15 (7.7)	0.033
Anti-Jo-1 antibody	59 (10.9)	45 (13.1)	14 (7.1)	0.033
Anti-SRP antibody	77 (14.3)	40 (11.7)	37 (18.9)	0.021
Anti-PL-7 antibody	40 (7.4)	30 (8.7)	10 (5.1)	0.120
Anti-PL-12 antibody	20 (3.7)	11 (3.2)	9 (4.6)	0.413
Anti-EJ antibody	24 (4.5)	21 (6.1)	3 (1.5)	0.013
Anti-OJ antibody	9 (1.7)	7 (2.0)	2 (1.0)	0.304
Anti-ARS antibody	145 (26.9)	110 (32.1)	35 (17.9)	< 0.001

Values are presented as number (%) or median (range).

IIM-ILD, idiopathic inflammatory myopathy patients with interstitial lung disease; IIM-NILD, idiopathic inflammatory myopathy patients without interstitial lung disease; ESR, erythrocyte sedimentation rate; CRP, C-reactive protein; NLR, neutrophil-to-lymphocyte ratio; PLR, platelet-lymphocyte ratio; CAR, the C-reactive protein to Albumin ratio; EAR, the erythrocyte sedimentation rate to Albumin ratio; ARS, aminoacyl-tRNA synthetase.

### Laboratory findings in IIM-ILD and IIM-NILD

As for laboratory findings, IIM-ILD patients had higher values of PLR (*p* = 0.013), GLO (*p* < 0.001), CRP (*p* < 0.001), ESR (*p* < 0.001), CAR (*p* < 0.001), EAR (*p* < 0.001) and ferritin (*p* < 0.001), except for ALB levels (*p* < 0.001). For the myositis-associated autoantibodies (MAA), IIM-ILD patients were prone to occur anti-Ro52 antibodies positive. Unsurprisingly, there was a significant difference between these two groups in terms of MSA. The IIM-ILD group showed more anti-aminoacyl-tRNA synthetase (anti-ARS) and anti-MDA5 antibodies positive. However, the IIM-NILD group had more anti-TIF1-γ, anti-Mi-2α, anti-NXP2, and anti-SRP antibodies positive (see Supplementary Fig. S1 online).

### Risk factors independently associated with IIM-ILD and IIM-NILD patients

To determine the independent risk factors of ILD in IIM patients, we employed univariate and multivariate logistic regression analyses combined with clinical applications. CAR was confirmed as a significant risk factor of ILD in multivariable analysis, increasing the risk by 55.4% (OR = 1.554, 95% CI: 1.091–2.212, *p* = 0.014). Other factors associated with an increased risk of mortality included the age at diagnosis (OR = 1.029, 95% CI: 1.010–1.047, *p* = 0.002), arthralgia (OR = 1.954, 95% CI: 1.144–3.339, *p* = 0.014), lung infection (OR = 1.803, 95% CI: 1.068–3.046, *p* = 0.027), hemoglobin (OR = 1.016, 95% CI: 1.002–1.031, *p* = 0.022), anti-ARS antibody positive (OR = 2.830, 95% CI: 1.673–4.787, *p* < 0.001) and anti-MDA5 antibody positive (OR = 7.472, 95% CI: 3.914–14.263, *p* < 0.001) (Table 2).

**Table 2:** Risk factors of ILD in patients with idiopathic inflammatory myopathies.

Covariate	Adjusted OR (95% CI)	*p* value
Age at diagnosis	1.029 (1.010–1.047)	0.002
Female	1.237 (0.757–2.021)	0.396
Gottron’s rash	1.138 (0.693–1.867)	0.610
Arthralgia	1.954 (1.144–3.339)	0.014
Mechanic’s hand	4.365 (0.946–20.144)	0.059
Dysphagia	0.717 (0.406–1.268)	0.253
Tumor	0.615 (0.280–1.351)	0.226
Lung infection	1.803 (1.068–3.046)	0.027
Tuberculosis	0.512 (0.229–1.143)	0.102
Respiratory failure	2.379 (0.716–7.910)	0.157
Hemoglobin	1.016 (1.002–1.031)	0.022
Ferritin	1.000 (1.000–1.001)	0.799
CAR	1.554 (1.091–2.212)	0.014
NLR	0.980 (0.919–1.046)	0.545
PLR	1.001 (0.999–1.002)	0.540
Anti-Ro52 antibody	1.441 (0.931–2.231)	0.101
Anti-ARS antibody	2.830 (1.673–4.787)	< 0.001
Anti-MDA5 antibody	7.472 (3.914–14.263)	< 0.001

CAR, the C-reactive protein to Albumin ratio; NLR, Neutrophil-to-lymphocyte ratio; PLR, platelet-lymphocyte ratio; ARS, aminoacyl-tRNA synthetase.

Further analysis compared the value of CAR between the IIM-ILD group and the IIM-NILD group. As expected, the value of CAR was higher in the IIM-ILD group compared with the IIM-NILD group (see Supplementary Fig. S2a online). Using receiver operating characteristic (ROC) analysis, The area under the ROC curve (AUC) was 0.6589 (95% CI: 0.6108–0.7070, *p* < 0.001) (see Supplementary Fig. S2b online). An optimal cutoff value for the CAR was derived from the point with the maximum Youden index. This cutoff point for CAR was 0.0680, yielding 83.7% sensitivity and 43.9% specificity.

### Comparison of clinical features of survivors and decedents in IIM-ILD

IIM-ILD patients were followed up, and 38 of the 343 patients with IIM-ILD were lost during follow-up. The follow-up period for all patients ranged from 1 to 78 months, with a median follow-up of 26 months. Baseline characteristics were compared among patients between the survival and the decedent groups. Of the 72 dead patients, 68.1% (49/72) of them were diagnosed with DM, and 6.9% (5/72) were diagnosed with PM (Table 3). The average age of the patients who died was higher than that of the survivors (*p* < 0.001), and the decedents had a higher rate of patients presented at the onset of ILD (*p* = 0.003) and shorter disease duration than the survivors (*p* = 0.018). Concerning clinical symptoms, chest tightness, dyspnea, dry mouth and fever were significantly more prevalent in the decedents compared with the survivors, while no statistical difference was found regarding the skin, joint and muscle manifestations. As for the comorbidities, the decedents were more likely to combine with lung infection (*p* = 0.002) and respiratory failure (*p* < 0.001). Compared with the survivors, the decedents had lower values of serum hemoglobin (*p* < 0.001) and ALB (*p* < 0.001). Moreover, NLR (*p* < 0.001), PLR (*p* = 0.001), CRP (*p* < 0.001), ESR (*p* = 0.002), CAR (*p* < 0.001), EAR (*p* < 0.001) and ferritin (*p* < 0.001) were significantly higher in the decedents compared with the survivors. In addition, we compared antibodies between the survivors and the decedents, and no differences were found regarding anti-Ro52 antibodies. The decedents showed a higher positive rate of anti-MDA5 antibodies (66.1% vs. 44.6%, *p* = 0.014) and lower positive rate of anti-Jo-1 antibodies (5.6% vs.15.0%, *p* = 0.036) and anti-SRP antibodies (4.2% vs.13.7%, *p* = 0.026) (Table 3).

**Table 3:** Comparison of clinical characteristics between survivors and decedents group in IIM-ILD patients.

Characteristic	Survivors	Decedents	*p*-value
Number	233 (76.4)	72 (23.6)	
Age at diagnosis (years)	52.03 ± 10.98	58.57 ± 11.17	< 0.001
Duration of disease, (years)	4 (2–11.5)	2 (1–6)	0.018
Female	151 (64.8)	44 (61.1)	0.568
Current smokers	22 (9.4)	6 (8.3)	0.497
ILD-onset	72 (30.9)	36 (50.0)	0.003
Diagnosis			0.135
Dermatomyositis	125 (53.6)	49 (68.1)	
Polymyositis	32 (13.7)	5 (6.9)	
Department			< 0.001
Respiratory	38 (16.3)	32 (44.4)	
Rheumatology	169 (72.5)	28 (38.9)	
Dermatology	18 (7.7)	8 (11.1)	
Signs			
Thick breath sounds	59 (25.3)	27 (37.5)	0.033
Low breath sounds	19 (8.2)	12 (16.7)	0.035
Moist rales	118 (50.6)	54 (75.0)	< 0.001
Clinical manifestation			
Respiratory symptoms			
Cough	126 (54.1)	48 (66.7)	0.059
Expectoration	91 (39.1)	35 (48.6)	0.150
Chest tightness	28 (12.0)	17 (23.6)	0.015
Dyspnea	129 (55.4)	53 (73.6)	0.006
Skin manifestations			
Gottron’s rash	99 (42.5)	38 (52.8)	0.125
Raynaud’s phenomenon	19 (8.2)	4 (5.6)	0.465
Mechanic’s hand	21 (9.0)	7 (9.7)	0.855
Joint manifestations			
Arthralgia	92 (39.5)	24 (33.3)	0.347
Joint tenderness	49 (21.0)	8 (11.1)	0.059
Swollen joint	45 (19.3)	7 (9.7)	0.059
Muscle manifestations			
Myalgia	99 (42.5)	26 (36.1)	0.336
Myasthenia	171 (73.4)	50 (69.4)	0.512
Dysphagia	31 (13.3)	15 (20.8)	0.119
Others			
Dry mouth	30 (12.9)	20 (27.8)	0.003
Fever	54 (23.2)	30 (41.7)	0.002
Hair loss	25 (10.7)	6 (8.3)	0.556
Weight loss	105 (45.1)	39 (54.2)	0.176
Comorbidities			
Tumor	9 (3.9)	5 (6.9)	0.356
Lung infection	88 (37.8)	42 (58.3)	0.002
Tuberculosis	10 (4.3)	5 (6.9)	0.358
Hypertension	39 (16.7)	13 (18.1)	0.460
Diabetes	18 (7.7)	7 (9.7)	0.589
Dyslipidemia	102 (43.8)	34 (47.2)	0.607
Respiratory failure	13 (5.6)	35 (48.6)	< 0.001
Laboratory results			
Hemoglobin (g/L)	123.8 ± 17.1	115.7 ± 15.3	< 0.001
Monocyte (*10^9^/L)	0.44 ± 0.25	0.40 ± 0.20	0.235
NLR	4.1371 (2.6994–6.8143)	7.2161 (3.7490–10.2477)	< 0.001
PLR	207.21 (141.37–303.83)	269.24 (184.74–382.21)	0.001
Albumin (g/L)	32.68 ± 5.55	27.79 ± 5.53	< 0.001
Globulin	29.8 (26.1–33.9)	31.0 (27.4–35.3)	0.108
Creatine kinase (U/L)	201.2 (56.6–1227.1)	106.9 (50.4–292.9)	0.036
Myohemoglobin (U/L)	117.1 (49.1–729.0)	106.8 (59.5–245.9)	0.411
CRP (mg/L)	5.38 (2.47–14.25)	14.65 (5.88–46.58)	< 0.001
ESR (mm/h)	31 (14–49)	38 (24–61)	0.002
CAR	0.1572 (0.0712–0.4880)	0.5299 (0.2170–1.6427)	< 0.001
EAR	0.8824 (0.4302–1.4838	1.3376 (0.8937–2.3218)	< 0.001
Ferritin (ng/ml)	394.2 (212.8–526.3)	534 (422.0–586.3)	< 0.001
Myositis associated autoantibodies			
Anti-Ro-52 antibody	139 (59.7)	50 (69.4)	0.135
Myositis specific autoantibodies			
Anti-MDA5 antibody	104 (44.6)	44 (66.1)	0.014
Anti-Jo-1 antibody	35 (15.0)	4 (5.6)	0.036
Anti-SRP antibody	32 (13.7)	3 (4.2)	0.026
Image abnormalities			
Irregular linear opacities	140 (60.1)	44 (61.1)	0.876
Grid shadow	71 (30.5)	27 (37.5)	0.264
Nodular shadow	98 (42.1)	24(33.3)	0.186
Septal thickening	59 (25.3)	20 (27.8)	0.678
Ground-glass opacities	76 (32.6)	22 (30.6)	0.743
Patch shadow	151 (64.8)	61 (84.7)	0.001
Traction bronchodilation	18(7.7)	9 (12.5)	0.213
Honeycombing	12 (5.2)	9 (12.5)	0.035
Pleural effusion	30 (12.9)	24 (33.3)	< 0.001
Therapy			
Corticosteroids	229 (98.3)	69 (95.8)	0.362
Immunosuppressants	198 (85.0)	36 (50.0)	< 0.001
Plasma exchange	15 (6.4)	17 (23.6)	< 0.001
IVIG	75 (32.2)	39 (54.2)	< 0.001
Pirfenidone	21 (9.0)	3 (4.2)	0.182

Values are presented as number (%) or median (range).

ILD, interstitial lung disease; ESR, erythrocyte sedimentation rate; CRP, C-reactive protein; NLR, Neutrophil-to-lymphocyte ratio; PLR, platelet-lymphocyte ratio; CAR, the C-reactive protein to albumin ratio; EAR, the erythrocyte sedimentation rate to Albumin ratio; IVIG, intravenous immunoglobulin.

The HRCT was available in all 305 IIM-ILD patients. The most common abnormality was patch shadow (212/305, 69.5%), and the second common abnormality was irregular linear opacities (184/305, 60.3%). Honeycombing was the least common HRCT presentation (21/305, 6.9%). The HRCT image of the decedents had more patch shadow (*p* = 0.001), honeycombing (*p* = 0.035), and pleural effusion (*p* < 0.001) than the survivors. The survivors were more likely to be treated with immunosuppressants, while the decedents were treated with plasma exchange and intravenous immunoglobulin (IVIG).

To explore the potential prognostic significance of these parameters, we analyzed the relationship between these parameters and survival in combination with clinical applications.

### Significance of parameters and cut-off determination in prognosis

**Figure 1:**
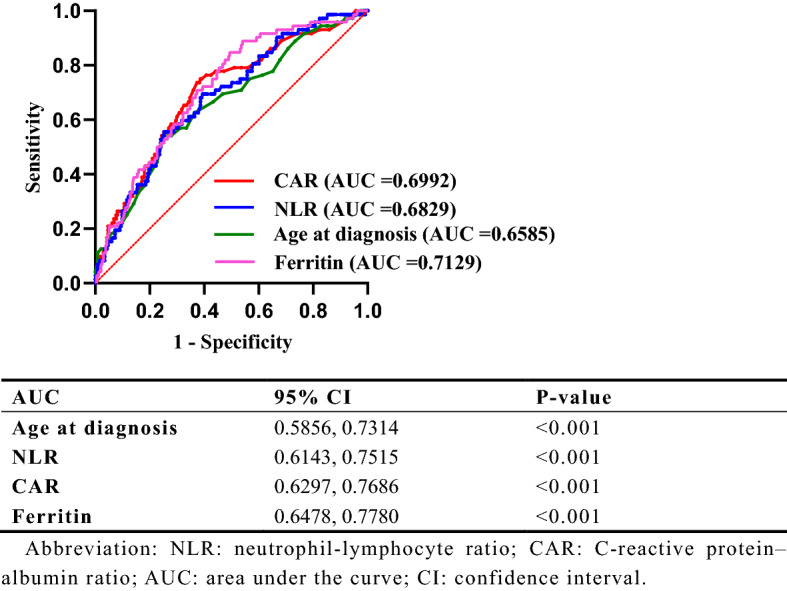
Receiver operator characteristic curves for predicting mortality in idiopathic inflammatory myopathy-associated interstitial lung disease (IIM-ILD). NLR, neutrophil–lymphocyte ratio; CAR, C-reactive protein–albumin ratio; AUC, area under the curve; CI, confidence interval.

Since age at diagnosis, NLR, CAR and ferritin were significantly associated with mortality in the patients with IIM-ILD, we chose the variables with *p* values < 0.05 to perform cut-off optimization for predicting mortality in our research. Optimized cut-offs for each variable were determined using standard ROC curve analysis. In ROC curve analyses, the AUCs for mortality of IIM-ILD were 0.6585 (*p* < 0.001), 0.6829 (*p* < 0.001), 0.6992 (*p* < 0.001) and 0.7129 (*p* < 0.001) for age at diagnosis, NLR, CAR and ferritin, respectively. Using the ROC curves, optimized cut-offs of the age at diagnosis (59.5), NLR (6.6109), CAR (0.2506) and ferritin (397.68) for IIM-ILD patients. The sensitivity and specificity were 54.2% and 75.1% for the age at diagnosis, 55.6% and 74.7% for the NLR, 73.6% and 63.9% for the CAR, and 84.7% and 50.6% for the ferritin (Fig. 1).

### Survival analysis of patients in IIM-ILD

**Figure 2:**
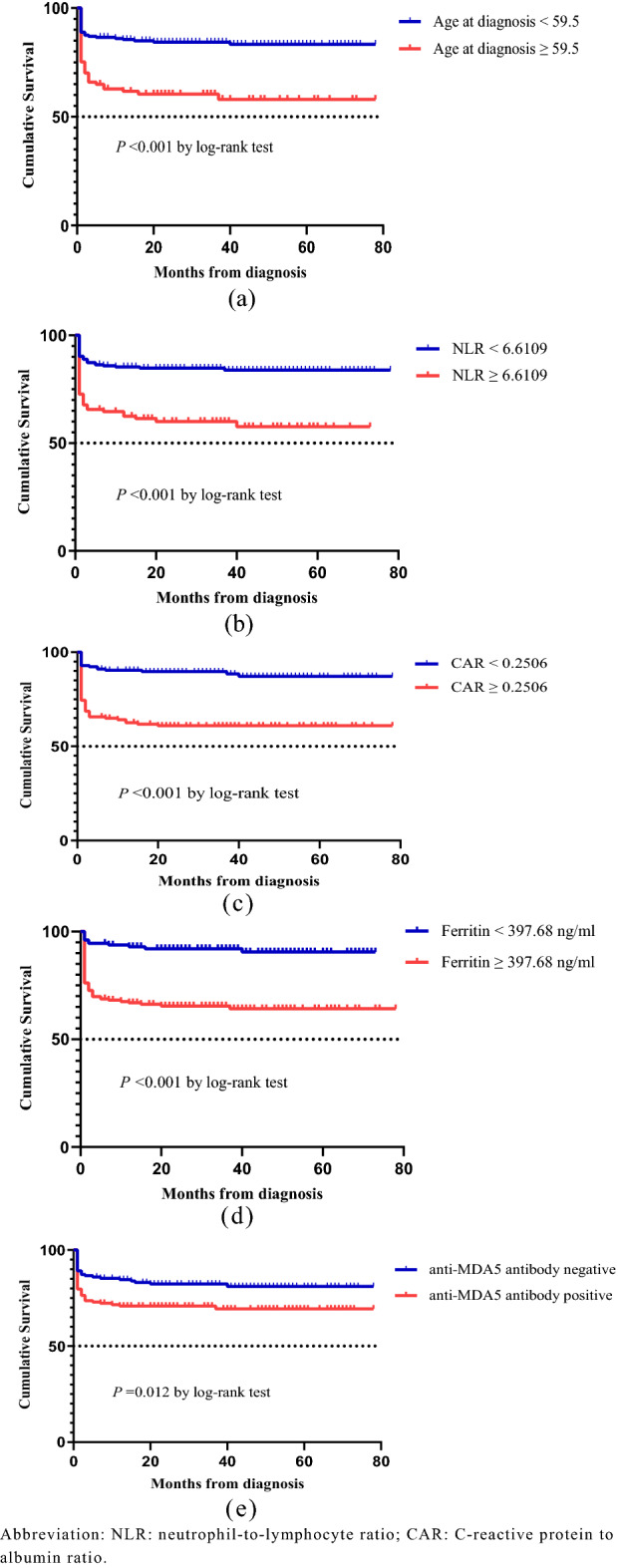
Survival curves for overall survival in patients with idiopathic inflammatory myopathy—associated Interstitial lung disease, stratified by age at diagnosis (**a**), NLR (**b**), CAR (**c**), ferritin (**d**) and anti-MDA5 antibody positive (**e**). NLR, neutrophil-to-lymphocyte ratio; CAR, C-reactive protein to albumin ratio.

The results of multivariable Cox regression analyses of factors associated with mortality are presented in Table 4. Cox regression analyses showed that age at diagnosis ≥ 59.5 (HR = 2.673, 95% CI: 1.588–4.499, *p* < 0.001), NLR ≥ 6.6109 (HR = 2.004, 95% CI: 1.193–3.368, *p* = 0.009), CAR ≥ 0.2506 (HR = 1.864, 95% CI: 1.041–3.339, *p* = 0.036), ferritin ≥ 397.68 (HR = 2.451, 95% CI: 1.245–4.827, *p* = 0.009) and anti-MDA5 antibody (HR = 1.928, 95% CI: 1.123–3.309, *p* = 0.017) were significant risk factors for all-cause mortality in IIM-ILD patients (Table 4). Moreover, significantly lower cumulative survival was observed in IIM-ILD patients with higher age at diagnosis (Fig. 2a), NLR (Fig. 2b), CAR (Fig. 2c), ferritin (Fig. 2d) and anti-MDA5 antibody positive (Fig. 2e).

**Table 4:** Results of multivariable Cox regression analyses for mortality IIM-ILD.

Variables	HR	95% CI	*p*-value
Age at diagnosis ≥ 59.5	2.673	1.588, 4.499	< 0.001
Female	0.786	0.474, 1.303	0.351
NLR ≥ 6.6109	2.004	1.193, 3.368	0.009
CAR ≥ 0.2506	1.864	1.041, 3.339	0.036
Ferritin ≥ 397.68	2.451	1.245, 4.827	0.009
Anti-MDA5 antibody	1.928	1.123, 3.309	0.017
Anti-Jo-1 antibody	0.477	0.169, 1.345	0.162
Honeycombing	1.798	0.883, 3.664	0.106

NLR, neutrophil-to-lymphocyte ratio; CAR, C-reactive protein to albumin ratio.

## Discussion

This present study reviewed the demographic, clinical, and laboratory characteristics as well as outcomes of IIM-ILD. We investigated independent risk factors of ILD and predictors of IIM-ILD patients. In our cohort, 343 (63.6%) IIM patients presented ILD, the incidence of ILD in IIM patients was close to 20% to 78%, as in previous studies^6–8^. Considering the high incidence of ILD in IIM, HRCT is essential, which has been proven as the pivotal radiologic evaluation in ILD because of its greater sensitivity compared with chest radiography, especially for early changes^17^. Our study identified arthralgia and lung infection as risk factors for ILD. Patients presenting with arthralgia tend to actively seek medical advice from rheumatology, which may lead to early recognition and diagnosis of ILD in IIM patients. However, the lack of specificity of arthralgia may experience delayed diagnosis and treatment of the disease due to neglect by patients and other departments. Patients may only present to respiratory departments when there is significant dyspnoea or even respiratory failure, thus missing the opportunity for early treatment. So we should strengthen the early recognition of patients’ symptoms to reduce the rate of missed diagnosis or misdiagnosis. Lung infection may cause direct damage to the lung, which in turn activates abnormal damage repair and ultimately leads to the development of pulmonary fibrosis. In addition, infection causes immune-mediated lung injury, activating immune cells such as macrophages, neutrophils, eosinophils and Th2 cells, which secrete large amounts of pro-inflammatory and pro-fibrotic factors, causing persistent lung damage and ultimately leading to pulmonary fibrosis^18^. Vaccination would play a key role in preventing lung infection and probably flares of ILD and mortality, we will explore the relationship between vaccines and mortality in patients with IIM-ILD in the future study. This study focuses on CAR, an indicator of the balance between CRP and ALB^19^. CAR is a newly discovered prognostic indicator of inflammation, which has been shown to have predictive value in neurocritical illness^20^, transcatheter aortic valve replacement^21^, neoplasms^22^, and sepsis^23^. It can be used as a predictor of disease severity in lung disease, including pneumonia^24^, lung cancer^25^, COPD^26^, and other diseases, but its application in ILD is rare. CRP is a marker of systemic inflammation, and ALB is frequently included as an indicator of frailty and nutrition. CRP levels increase, and ALB levels decrease due to inflammation depletion. It has been reported that IL-6 can affect both CRP and ALB^27,28^, IL-6 has been proven that involved in the development and progression of fibrosis, so the prognosis of IL-6 in IIM-ILD needs to be further studied. Although some studies have shown that ALB^9^ or CRP^29^ can be used as predictors of IIM, few studies have certified CAR as a predictor of IIM-ILD. CAR is superior to either CRP or ALB alone in predicting prognosis for patients with acute medical conditions^30^, which suggests that this biomarker may reflect parenchymal lung inflammation leading to irreversible injury. In this study, both univariate and multivariate logistic regression analyses showed that CAR was significantly associated with the occurrence of ILD in IIM. In addition, COX regression demonstrated that CAR was a strong predictor of the prognosis of IIM-ILD. Kaplan–Meier curve showed that the mortality rate in the CAR < 0.2506 group was higher than that in the CAR ≥ 0.2506 group. Although our data show relatively low sensitivity and specificity of CAR in predicting mortality in IIM-ILD patients, all these results suggest that CAR may be a suitable prognostic biomarker for patients with IIM and can even be used to assess nutritional status in patients with IIM. It is worth further studying its application value in IIM, considering its cost-practical value.

As for MSA, It is well known that the incidence of ILD is closely related to the anti-ARS and anti-MDA5 antibodies. Anti-ARS antibodies are reported be detected in 20–40% of patients with IIM^31,32^ and the incidence of ILD in anti-ARS antibody-positive patients is 79–95%^33–35^. In the current study, 145 (26.9%) patients were tested for the anti-ARS antibody, and 76% (110/145) of the patients showed ILD, which is in line with previous estimates. Our analysis also proved that it is a risk factor for ILD. The anti-MDA5 antibody has been regarded as a vital serum predictive marker for IIM, often indicative of a poor prognosis of IIM^36–39^. Early diagnosis and more effective treatment are essential, so we should pay more attention to the patients with anti-MDA5 antibody positive and give them aggressive treatment.

About MAA, Gui et al.^40^showed that the anti-Ro52 antibody could predict the prognosis of IIM-ILD, but this study did not verify its role as a predictor of IIM-ILD. Given that the anti-Ro52 antibody often combines with other MSA antibodies positive, it was difficult to determine the 'target antibody' in duo-positive or multi-positive patients. A real-world Australian study^41^did not support a prognostic role for the anti-RO52 antibody in IIM-ILD. Its prophetic role in IIM-ILD is still controversial^42,43^. Currently, no clear guideline or consensus exists, and more extensive prospective studies are needed for validation.

The older the age at diagnosis, the worse the prognosis, which may be related to the fact that elderly patients are prone to immune disorders, and their lung function becomes worse with age. This study also collected data related to pulmonary function tests (PFT). However, given that most patients were treated in the rheumatology and dermatology department, there were too much missing data on PFT, which was not included in the discussion. In the subsequent study, we will further supplement the data related to PFT. NLR has been recognized as a practical and valuable prognostic biomarker. It can be used as a prognostic indicator for COVID-19^44^, systemic sclerosis^45^, spinal arthritis^46^, malignant neoplasms^47^, idiopathic pulmonary fibrosis^48^ and other diseases. Regarding the use of NLR in IIM, a retrospective study in South Korea has demonstrated the predictive role of NLR in IIM-ILD, with higher values associated with a worse prognosis^49^. The optimal cut-off value for this study was 4.775, while our study demonstrated that the optimal cut-off was 6.6109. Studies have suggested that ferritin is associated with the prognosis of IIM-ILD patients^50,51^. Nevertheless, our study with a larger sample size showed that serum ferritin was a risk factor for poor prognosis, thus providing more parameters for evaluating the prognosis of IIM-ILD patients. However, different researches have different cut-off values of ferritin for prognosis. Ferritin showed a lower value in our study than in others. This difference may be due to the different populations and the testing methods.

This study had some limitations. Firstly, as this study is retrospective, there will inevitably be information and recall bias. Secondly, our follow-up method, mainly through telephone follow-up, could not obtain the treatment method for the whole follow-up period, so the impact of treatment on prognosis could not be evaluated. Finally, there were some truncated data in the survival analysis, so we need to further track the survival status of these patients in the subsequent time.

## Conclusion

IIM with elevated CAR and the anti-MDA5 antibody positive are more likely to occur ILD and have an increased risk of death. In clinical application, CAR is a readily accessible measurement that can be incorporated into the risk assessment of patients considering IIM. For future studies, our study may help provide longitudinal information on prognosis, survival, and mortality in patients with IIM.

## Supplementary Information

Supplementary Figures.

## Data Availability

The datasets used and/or analysed during the current study are available from the corresponding author on reasonable request.
