# Supplementary material for: The prognostic role of C-reactive protein to albumin ratio and anti-MDA5 antibody-positive in idiopathic inflammatory myopathy: a retrospective study
Source: Sci Rep. 2023 Mar 8;13:3863. doi: 10.1038/s41598-023-30595-y (PMC9992913; doi:10.1038/s41598-023-30595-y)
Supplement: Supplementary file 1 — Supplementary Figures. [file 41598_2023_30595_MOESM1_ESM.docx]

**The prognostic role of C-reactive protein to albumin ratio and anti-MDA5 antibody-positive in idiopathic inflammatory myopathy: a retrospective study**

Pei Zhou1; Qinxue Shen1; Shiting Zhou1; Xiaoli Ouyang1; Ting Guo1; Min Song1; Wei Guo1; Yi Zhang1; Hong Peng1*

1 Division of Pulmonary and Critical Care Medicine, The Second Xiangya Hospital, Central South University, Changsha, Hunan 410011, China

* Corresponding author: Hong Peng Email: penghong66@csu.edu.cn

**Figure S1.** Myositis-specific autoantibodies (MSAs) distribution of the 539 patients with idiopathic inflammatory myopathies.


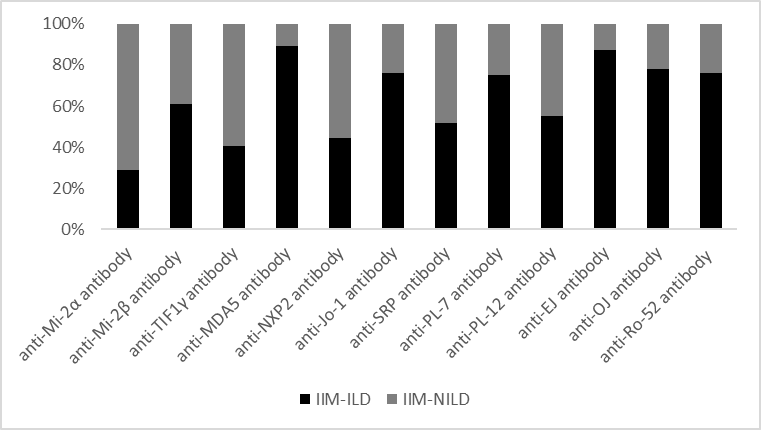


Abbreviation: IIM: Idiopathic inflammatory myopathy; IIM-ILD: Idiopathic inflammatory myopathy-associated interstitial lung disease; IIM-NILD: Idiopathic inflammatory myopathy without interstitial lung disease.

**Figure S2.** The value of CAR for discriminating IIM-ILD from IIM-NILD.

(a)

(b)

Abbreviation: IIM-ILD: Idiopathic inflammatory myopathy-associated interstitial lung disease; IIM-NILD: Idiopathic inflammatory myopathy without interstitial lung disease; AUC: area under the curve.
